# Associations of Hospital and Patient Characteristics with Fluid Resuscitation Volumes in Patients with Severe Sepsis: Post Hoc Analyses of Data from a Multicentre Randomised Clinical Trial

**DOI:** 10.1371/journal.pone.0155767

**Published:** 2016-05-19

**Authors:** Peter Buhl Hjortrup, Nicolai Haase, Jørn Wetterslev, Anders Perner

**Affiliations:** 1 Department of Intensive Care, Copenhagen University Hospital, Rigshospitalet, Blegdamsvej 9, 2100, Copenhagen, Denmark; 2 Copenhagen Trial Unit, Copenhagen University Hospital, Rigshospitalet, Blegdamsvej 9, 2100, Copenhagen, Denmark; 3 Centre for Research in Intensive Care, Copenhagen, Denmark; Hospital Sirio-Libanes, BRAZIL

## Abstract

**Purpose:**

Fluid resuscitation is a key intervention in patients with sepsis and circulatory impairment. The recommendations for continued fluid therapy in sepsis are vague, which may result in differences in clinical practice. We aimed to evaluate associations between hospital and patient characteristics and fluid resuscitation volumes in ICU patients with severe sepsis.

**Methods:**

We explored the 6S trial database of ICU patients with severe sepsis needing fluid resuscitation randomised to hydroxyethyl starch 130/0.42 vs. Ringer’s acetate. Our primary outcome measure was fluid resuscitation volume and secondary outcome total fluid input administered from 24 hours before randomisation until the end of day 3 post-randomisation. We performed multivariate analyses with hospital and patient baseline characteristics as covariates to assess associations with fluid volumes given.

**Results:**

We included 654 patients who were in the ICU for 3 days and had fluid volumes available. Individual trial sites administered significantly different volumes of fluid resuscitation and total fluid input after adjusting for baseline variables (P<0.001). Increased lactate, higher cardiovascular and renal SOFA subscores, lower respiratory SOFA subscore and surgery were all independently associated with increased fluid resuscitation volumes.

**Conclusions:**

Hospital characteristics adjusted for patient baseline values were associated with differences in fluid resuscitation volumes given in the first 3 days of severe sepsis. The data indicate variations in clinical practice not explained by patient characteristics emphasizing the need for RCTs assessing fluid resuscitation volumes fluid in patients with sepsis.

## Introduction

For decades fluid resuscitation has been considered a pivotal intervention in the treatment of patients with sepsis and circulatory impairment. The hemodynamic consequences of sepsis are complex and several pathophysiological characteristics serve as rationale for fluid administration including dehydration, increased vascular permeability leading to decreased intravascular fluid volume [1] and decreased vascular tone [2]. However, fluid administration also has potential adverse effects including increasing tissue edema [3] and electrolyte derangements [4, 5].

The optimal volume of fluid and indications for fluid resuscitation in severe sepsis are not established. Fluid resuscitation guided by central venous pressure in the first six hours of septic shock was a part of the protocol in the landmark trial of Early Goal-Directed Therapy (EGDT) in sepsis by Rivers et al that showed significantly increased survival with EGDT [6]. Since then, three large-scale multicentre trials have reported no benefit of EGDT vs. standard care [7–9], but the use of resources was increased with EGDT [10]. Beyond the first six hours the international guidelines for fluid resuscitation are vague and ungraded due to lack of evidence; the 2012 recommendation for continued fluid therapy from the Surviving Sepsis Campaign states: “Fluid challenge technique be applied wherein fluid administration is continued as long as there is hemodynamic improvement either based on dynamic (e.g. change in pulse pressure, stroke volume variation) or static (e.g. arterial pressure, heart rate) variables” [11].

The lack of firm evidence and vague recommendations may infer differences in clinical practice. The aim of this study was to investigate whether hospital characteristics adjusted for patient baseline characteristics were associated with differences in fluid resuscitation volumes in a multicentre randomised clinical trial in patients with severe sepsis needing fluid resuscitation. Furthermore, we aimed to describe patient baseline characteristics’ association with fluid resuscitation volumes.

## Methods

This was a retrospective analysis of data from the Scandinavian Starch for Severe Sepsis and Septic Shock (6S) trial. In the 6S trial adult patients with severe sepsis and need of fluid resuscitation were randomised to resuscitation with hydroxyethyl starch (HES) 130/0.42 (Tetraspan, B Braun Medical AG, Melsungen, Germany) or Ringer’s acetate (Sterofundin, B Braun Medical). Patients were randomised in 26 ICUs in Denmark, Finland, Norway and Iceland between December 2009 and November 2011. All non-Danish hospitals were university hospitals and all ICUs were located at different hospitals. Exclusion criteria were: Age < 18 years, treatment with > 1000 ml of any synthetic colloid within the last 24 h prior to randomisation; any form of renal replacement therapy (RRT), severe hyperkalaemia (p-K > 6 mmol/l) within the last 6 h and acute burn injury > 10% of body surface area, previously randomised in the 6S trial, allergy towards HES or malic acid, liver or kidney transplantation during that hospitalization, intracranial bleeding within that hospitalization, enrolment into another ICU trial of drugs with potential action on circulation, renal function or coagulation, and withdrawal of active therapy. Appropriate approvals and written consent from patients and/or legal substitutes were obtained according to national laws. The 6S trial was approved by the Danish Committee on Health Research Ethics. Patient information in the 6S trial database was anonymized and de-identified. No additional patient data were obtained for the present study and an ethics approval for the present study was not required according to Danish law. The protocol and the outcomes have been published [12–14].

In the present study we included all patients randomised into the 6S trial who had available fluid data from 24 hours before randomisation until day 3 post-randomisation, i.e. patients that had not died, had not been discharged and had no missing daily fluid data until day 3 after randomisation. As this was a post hoc study, a convenience sample was used and a sample size calculation was not performed.

The primary outcome measure was fluid resuscitation volume and the secondary outcome measure was total fluid input. Fluid resuscitation volume was defined as all masked and open-label trial fluids given in the first 3 days after randomisation in the 6S trial combined with crystalloids (not including fluids with medication) and colloids given from 24 hours before randomisation (day 0). Open label trial fluid (Ringer’s acetate) was administered in case of the maximum dose of 33 ml/kg/24h masked trial fluid was exceeded. If a patient was withdrawn from the trial intervention, then all crystalloids (not including fluids with medication) and colloids given from time of withdrawal to the end of day 3 after randomisation were regarded as fluid resuscitation. Reasons for withdrawal were described in the published protocol [13]. Fluid data were registered daily in the 6S trial database with differentiation between trial fluids, crystalloids, colloids, glucose solutions, fluids with medication, nutrition, and blood products. Total fluid input was defined as the sum of all fluids, including the fluid resuscitation volume.

We decided *a priori* to include the following patient baseline characteristics in the multivariate analyses: Simplified Acute Physiology Score (SAPS II) [15], age, weight, highest lactate +/- 2 hours from randomisation, surgery performed prior to randomisation, allocation group (HES or Ringer’s acetate) and individual Sequential Organ Failure Assessment (SOFA) subscores (cardiovascular, renal, liver, coagulation and respiratory) [16]. The CNS SOFA subscore was not registered in the 6S trial and was not included in the present study. The included hospital characteristics were Danish hospital (yes/no), university hospital (yes/no) and individual hospitals with at least 25 patients randomised in the 6S trial. The manuscript was prepared according to the Strengthening the Reporting of Observational Studies in Epidemiology (STROBE) statement [17].

### Statistical analyses

We performed analyses using a generalised linear model with fluid volumes as outcome. The following model builds were used:

Model 1. Hospital characteristics:

Fluid volumes ~ Danish hospital (yes/no) + University hospital (yes/no) + patient baseline characteristics (in order to perform adjustment by trial site, trial sites with less than 25 randomised patients were combined into one site).

Model 2. Individual trial site (only trial sites with at least 25 randomised patients were included):

Fluid volumes ~ Trial site (as a factor) + patient baseline characteristics

Model 3. Patient baseline characteristics:

Fluid volumes ~ Patient baseline characteristics + Trial site (as factor where trial sites with less than 25 randomised patients were combined into one site in order to perform adjustment).

The patterns of residuals were evaluated for each analysis to ensure adequate goodness of fit. If > 5% of baseline values were missing, multiple imputations of the missing values were performed, which has been reported to reduce risk of bias [18]. For each missing baseline value 10 imputations were performed where the missing value was imputed from SAPS II, age, weight, highest lactate, surgery performed prior to randomisation, allocation (HES or Ringer’s acetate) and individual SOFA component subscores, Danish hospital, university hospital, mortality within 90 days of randomisation and any use of RRT within 90 days of randomization. Complete case analyses were also performed.

Fluids given in the 24 hours prior to randomisation might include fluids given in the general ward where fluid registration was not as meticulous as in the ICU. Therefore, we conducted a sensitivity analysis excluding fluids given prior to randomisation, and another sensitivity analysis including the patients who had been discharged or had died within the first 3 days of randomisation. To investigate the impact of patients who were withdrawn from the intervention during the first 3 days after randomisation, we performed sensitivity analyses excluding these patients. We chose *a priori* to include trial site as a fixed-effect variable. To investigate the impact of this choice, we performed mixed model analyses with trial site analysed as a random-effects variable.

Statistical analyses were performed using SAS version 9.4 (SAS institute Inc., Cary, NC, USA). Two-sided P-values of less than 0.05 were considered statistically significant.

## Results

**Fig 1:**
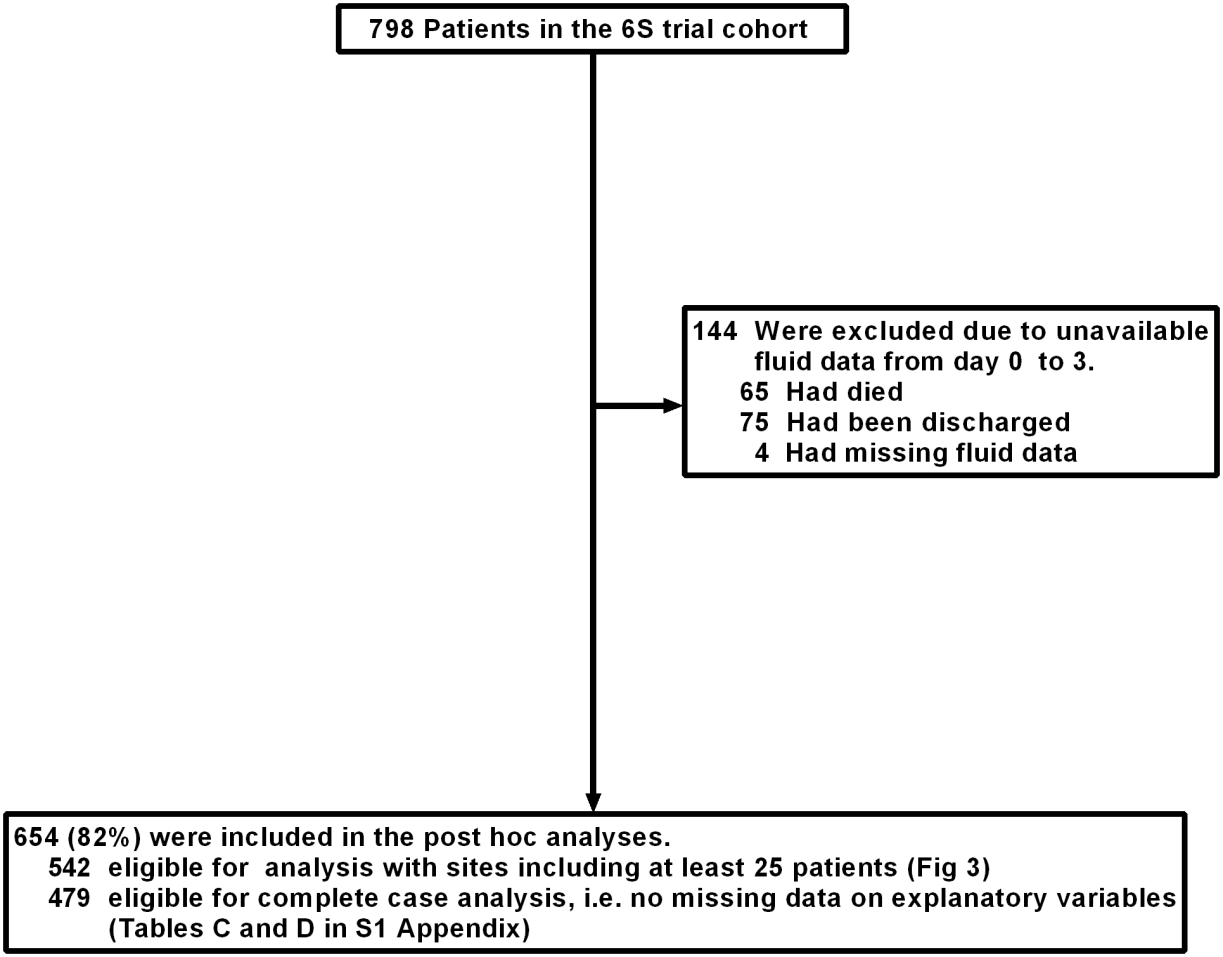
Flowchart of included patients.

Of the trial cohort of 798 patients, we included the 654 (82%) patients who had fluid volumes registered from day 0 to 3 (Fig 1). Of the 654 patients, 93 (14%) were withdrawed from the intervention during the first 3 days after randomisation resulting in 183/1962 (9%) of days where all administered crystalloids and colloids where included in the fluid resuscitation volume for the present analyses. Baseline characteristics are presented in Table 1.

**Table 1:** Patient baseline and hospital characteristics.

	Total cohort (n = 654)
Male	399 (61%)
Age (years)	66 (57–75)
SAPS II	50 (40–60)
Weight (kg)	78 (65–89)
Highest lactate +/- 2 hours from randomisation (mmol/l)	2.1 (1.4–3.4)
HES group	329 (50%)
Surgery prior to randomisation	232 (35%)
Source of sepsis	
- Lungs	369 (56%)
- Abdomen	209 (32%)
- Urinary tract	77 (12%)
- Soft tissue	74 (11%)
- Other source	59 (9%)
Hours from ICU admission to randomisation	4 (1–14)
SOFA score^1^	7 (5–9)
- Cardiovascular subscore	3 (1–4)
- Renal subscore	1 (0–2)
- Liver subscore	0 (0–1)
- Coagulation subscore	0 (0–1)
- Respiratory subscore	3 (2–3)
Danish hospital	583 (89%)
University hospital	321 (49%)
Randomised in trial site with ≥ 25 patients randomised (12 of 26 trial sites)	542 (83%)

All variables presented as median (interquartile range) or n (%). Individual SOFA subscores range from 0–4 with 4 being the most severe score.

^1^ The CNS component of the SOFA score was not reported in the 6S trial and is not included in the analysis.

Abbreviations: HES, hydroxyethyl starch. ICU, intensive care unit. SOFA, Sequential Organ Failure Assessment.

### Fluid volumes

**Fig 2:**
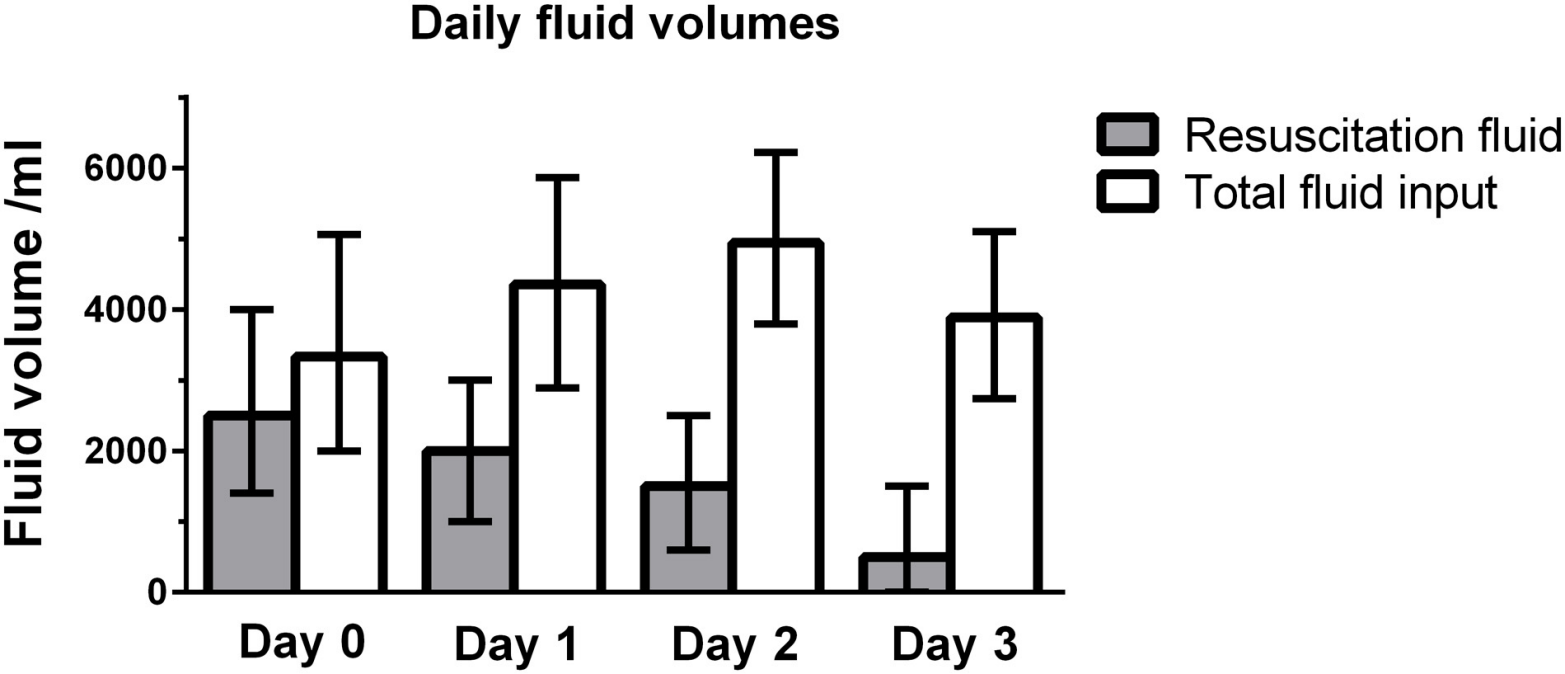
Daily fluid volumes presented as median with interquartile range (error bars). Resuscitation fluid was defined as crystalloids and colloids given from 24 hours prior to randomisation (day 0) until end of day 3 in the 6S trial.

The fluid resuscitation volume given from day 0 to day 3 was median 7,300 ml (interquartile range (IQR) 5,000–10,000 ml); total fluid input was median 16,912 ml (IQR 13,681–20,513 ml). Daily fluid volumes are presented in Fig 2. Fluids given with medication and nutrition combined constituted 72% of the difference between fluid resuscitation volume and total fluid input. The constituents daily of fluid input from day 0 to day 3 are presented in Table A in S1 Appendix.

### Hospital characteristics and fluid volumes

**Fig 3:**
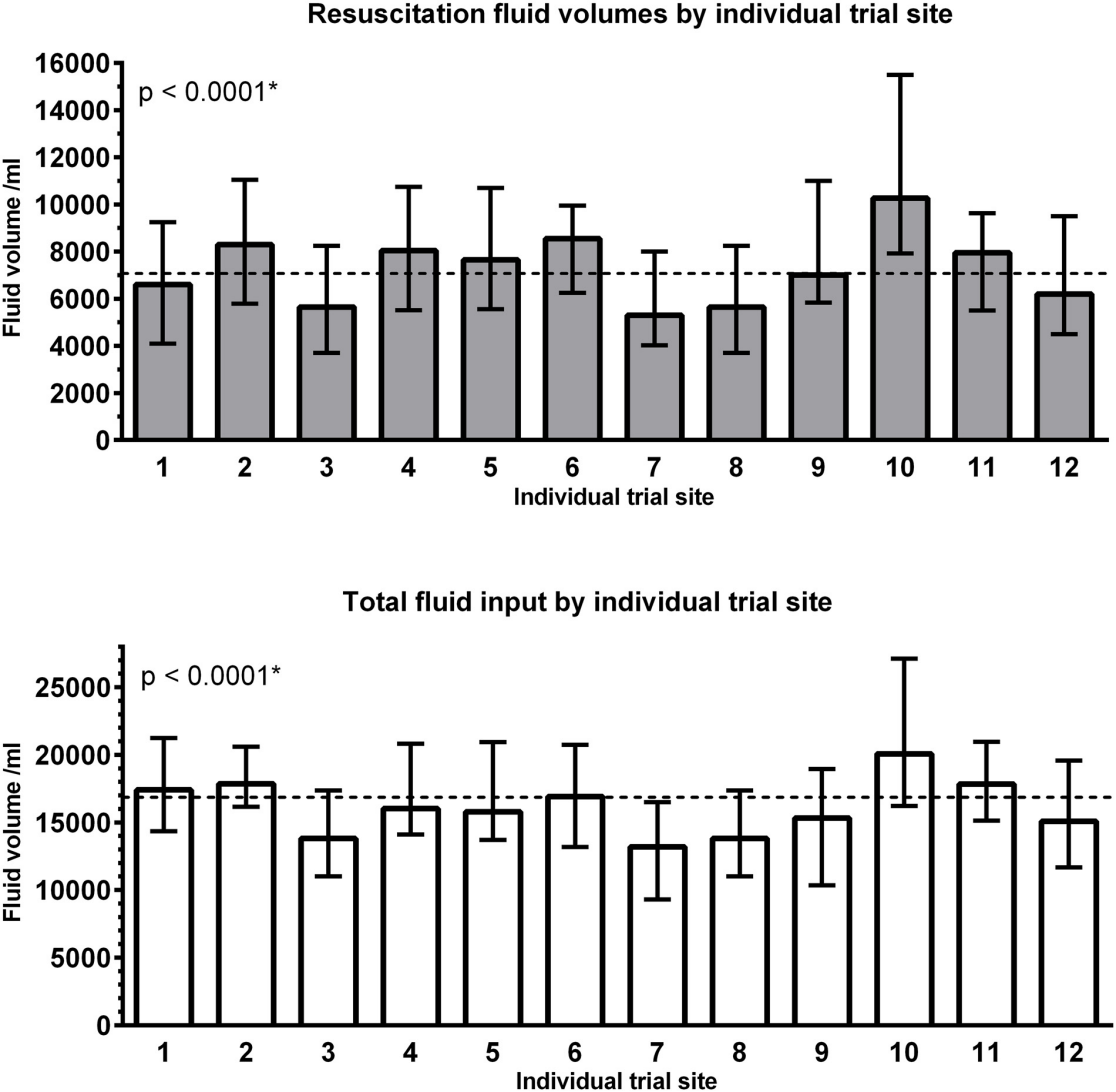
Resuscitation fluid volume (top panel) and total fluid input (bottom panel) by individual trial site with at least 25 randomised patients (n = 542). Fluid volumes presented as median with interquartile range (error bars). The horizontal dashed line denotes median for all patients. * P-value for trial site in a generalised linear model. Multiple generalised linear model build: Resuscitation fluid ~ Trial site (as a factor) + SAPS II + age + weight + highest lactate + surgery performed prior to randomisation (yes/no) + allocation (HES/Ringer’s acetate) + cardiovascular SOFA subscore + renal SOFA subscore+ liver SOFA subscore + coagulation SOFA subscore + respiratory SOFA subscore.

Individual trial sites administered significantly different fluid resuscitation volumes and total fluid input adjusted by patient baseline characteristics (p<0.001 for both outcome measures–Fig 3). Being admitted at a university hospital was associated with decreased fluid resuscitation volume, but no statistically significant difference in total fluid input (Table 2). Being admitted to a Danish hospital was associated with increased total fluid input, but no statistically significant difference in fluid resuscitation volumes. Unadjusted analyses are presented in Table B in S1 Appendix.

**Table 2:** Associations between hospital characteristics and fluid volumes given from day 0 to day 3. (n = 654).

	Resuscitation fluid (95% CI) (ml)	P-value	Total fluid input (95% CI) (ml)	P-value
Danish hospital vs. non-Danish hospital	-641 (-1787–506)	0.27	1773 (314–3232)	0.02
University hospital vs. non-university hospital	-825 (-1500– -150)	0.02	-618 (-1604–368)	0.22

Abbreviations: CI, confidence interval. HES, hydroxyethyl starch. SOFA, Sequential Organ Failure Assessment.

Multivariate generalised linear model build:

Fluid volume ~ Danish hospital (yes/no) + University hospital (yes/no) + SAPS II + age + weight + highest lactate + surgery performed prior to randomisation (yes/no) + allocation (HES/Ringer’s acetate) + cardiovascular SOFA subscore + renal SOFA subscore+ liver SOFA subscore + SOFA subscore + coagulation + respiratory SOFA subscore

### Patient baseline characteristics and fluid volumes

In the multivariate generalised linear model, increased lactate, increased cardiovascular and renal SOFA subscores, decreased respiratory SOFA subscore and having surgery performed prior to randomisation were all independently associated with increased fluid resuscitation volume and total fluid input (Table 3). In addition weight and increased coagulation SOFA subscore were associated with increased fluid input. Unadjusted analyses are presented in Table B in S1 Appendix.

**Table 3:** Associations between patient baseline characteristics and fluid volumes given from day 0 to day 3. (n = 654).

	Resuscitation fluids (95% CI) (ml)	P-value	Total fluid input (95% CI) (ml)	P-value
Age per year	-7 (-34–20)	0.60	-24 (-62–14)	0.21
SAPS II per unit	11 (-13–36)	0.35	14 (-24–51)	0.48
Weight per kg	15 (-0.3–31)	0.055	41 (18–64)	0.0005
Highest lactate per mmol/l	193 (53–332)	0.0069	289 (77–502)	0.008
Surgery vs. No surgery	1376 (732–2019)	< 0.0001	2941 (2006–3877)	< 0.0001
HES vs. Ringer’s	-276 (-844–292)	0.34	305 (-518–1129)	0.47
SOFA subscores per unit				
- Cardiovascular subscore	375 (184–566)	0.0001	632 (340–924)	< 0.0001
- Renal subscore	269 (11–528)	0.04	353 (8–698)	0.045
- Coagulation subscore	63 (-232–357)	0.68	885 (454–1315)	< 0.0001
- Liver subscore	-43 (-460–374)	0.84	61 (-541–663)	0.84
- Respiratory subscore	-397 (-729 –-65)	0.02	-215 (-676–245)	0.36

Individual SOFA subscores range from 0–4 with 4 being the most severe score. CNS component of the SOFA score was not reported in the 6S trial and is not included in the analysis.

Abbreviations: CI, confidence interval. HES, hydroxyethyl starch. ICU, intensive care unit. SOFA, Sequential Organ Failure Assessment.

Multivariate generalised linear model build:

Fluid volume ~ SAPS II + age + weight + highest lactate + surgery performed prior to randomisation (yes/no) + allocation (HES/Ringer’s acetate) + cardiovascular SOFA subscore + renal SOFA subscore+ liver SOFA subscore + coagulation SOFA subscore + respiratory SOFA subscore + trial site (as a factor, with hospital with less than 25 patients grouped)

### Missing data

Only the SAPS II (25% missing) needed multiple imputations. Complete case analyses did not change results noticeably (Tables C and D in S1 Appendix).

### Sensitivity analyses

When excluding fluids given prior to randomisation there was no difference in fluid resuscitation volumes between university hospitals and non-university hospitals (Table E in S1 Appendix). Also, the cardiovascular SOFA subscore, the respiratory SOFA subscore and having surgery performed were no longer associated with differences in fluid resuscitation volume (Table F in S1 Appendix).

When including patients who had been discharged or had died within three days of randomisation, higher lactate and being admitted to a non-university hospital were not associated with increased fluid resuscitation volume with statistical significance in contrast to the primary analyses (Tables G and H in S1 Appendix).

The analyses with trial site as a random-effects variable (Tables I and J in S1 Appendix) and the sensitivity analyses excluding patients who were withdrawn from the intervention did not change the results noticeably (Tables K and L in S1 Appendix).

## Discussion

In this post hoc analysis of data from the 6S trial we found that individual trial sites adjusted for patient baseline characteristics were associated with differences in administered fluid volumes in ICU patients with severe sepsis. Geographical differences in choice of fluid type have previously been reported [19], and the findings of the present study indicate differences in clinical practice not explained by patient characteristics and support our hypothesis that the vague recommendations on continued fluid therapy in sepsis result in differences in fluid therapy.

Patient baseline characteristics were associated with differences in fluid resuscitation volume. The association with fluid resuscitation volume differed between SOFA subscores. Thus, the association between combined SOFA score and fluid resuscitation volume may depend on the constitution of the SOFA subscores. In line with this, we found the SAPS II not to be statistically significantly associated with increased fluid resuscitation volume in either the adjusted or the unadjusted analysis. The SOFA score and the SAPS II are frequently used in multivariate analyses when adjusting for ‘illness severity’, but our data indicate that results must be interpreted with caution considering fluid volumes in sepsis. We unexpectedly found patient weight not to be statistically significantly associated with fluid resuscitation volume in either the multivariate or the univariate analysis, and the observed point estimate of 15 ml/kg difference was lower than expected. Recommendations for the initial fluid resuscitation are based on the baseline weight of the patient [11], and although the recommendations for continued fluid therapy do not explicitly mention weight, we had expected higher baseline weight to be associated with increased fluid resuscitation volume. From a physiological point of view it seems rational to increase fluid resuscitation volume with higher baseline weight due to the increased volume of distribution. Baseline weight, however, was associated with increased total fluid input.

Our finding that increased respiratory SOFA subscore was associated with less fluid resuscitation volume was in accordance with the results of the FACCT trial suggesting benefit of a conservative fluid strategy in patients with acute lung injury [20]. Our findings are also consistent with recent studies that found low blood pressure and increasing vasopressor dose (determinants of the cardiovascular SOFA subscore) to be the most frequent indications for fluid bolus in severe sepsis [21, 22]; oliguria (one of the determinants of renal SOFA subscore) was also a frequently used indication for fluid bolus in both studies. Of note, in the sensitivity analysis excluding fluids given prior to randomisation the cardiovascular SOFA subscore at baseline was not independently associated with increased fluid resuscitation volumes. Although these indications for fluid administration are frequent, it is still not established whether patients with severe sepsis and septic shock will benefit from receiving additional fluids. Alternative actions include continued/increased vasopressors or permitting wider physiological derangements [23]. The ability of treating clinicians to evaluate the balance between potential benefits and potential harms of fluid administration is impeded by the not yet fully understood hemodynamic consequences of giving fluids; a recent study found the relationship between cardiac output and mean arterial pressure in response to a fluid bolus to be unpredictable and inconsistent [24].

There was a marked difference in the results between resuscitation fluid volume and total fluid input for Danish hospitals vs. non-Danish, where Danish hospitals had higher total fluid input, but no difference in fluid resuscitation volume. This result may indicate regional difference in use of other fluids (e.g. fluids with medication and nutrition) in addition to the differences in fluid resuscitation volumes already described. Excluding fluids given prior to randomisation noticeably changed the results for the cardiovascular SOFA subscore and surgery. These findings indicate that these patient baseline characteristics primarily affected the fluid resuscitation administered in the early phase of septic shock, whereas the strong association between higher lactate at baseline and increased fluid resuscitation volume persisted when excluding fluids given prior to randomisation.

The present study had several strengths. First, the 6S trial had relatively detailed daily registration of fluid input, which enabled us to differentiate between types of administered fluids. Fluids given with nutrition and medication constitute a large proportion of the fluid input during the first days of severe sepsis, and we were able to exclude these non-resuscitation fluids in our analyses. Second, the 6S trial randomised patients in different types of hospitals in four countries and thus increasing the external validity of our results. Third, the meticulous registration of fluid data resulted in only 0.4% of patients eligible for this study had missing fluid data.

There were also limitations. First, patients who had died or had been discharged were not included in the present analyses, which decrease the generalizability of the results. However, the sensitivity analysis including these patients did not change the results noticeably. Second, it is possible that hospitals participating in a randomised clinical trial of fluid therapy (in this case type of fluid) may have a different clinical practice as compared to non-participating hospitals in Scandinavia, thus introducing a potential selection bias. Third, all non-Danish hospitals were university hospitals which makes analyses of potential interaction unfeasible. Fourth, although the statistical assessments of model fits were adequate, there may be clinical considerations when interpreting the results. Especially the renal SOFA subscore should be interpreted with caution as extreme values (e.g. established anuria) might have led the clinicians to withhold further fluid resuscitation.

## Conclusions

Hospital characteristics adjusted for patient baseline values were associated with differences in the administered fluid resuscitation volumes within the first 3 days of severe sepsis. Increased cardiovascular and renal SOFA subscores and decreased respiratory SOFA subscore at baseline were associated with increased fluid resuscitation volumes. Our data indicate variations in clinical practice not explained by patient characteristics emphasizing the need for RCTs of fluid resuscitation volumes in patients with severe sepsis.

## Supporting Information

S1 Appendix Supplemental tables and analyses. (DOCX)

S1 Dataset Dataset with multiple imputations. (SAS7BDAT)
