# Supplementary material for: Associations of Hospital and Patient Characteristics with Fluid Resuscitation Volumes in Patients with Severe Sepsis: Post Hoc Analyses of Data from a Multicentre Randomised Clinical Trial
Source: PLoS One. 2016 May 19;11(5):e0155767. doi: 10.1371/journal.pone.0155767 (PMC4873042; doi:10.1371/journal.pone.0155767)
Supplement: S1 Appendix — (DOCX) [file pone.0155767.s001.docx]

Supplement

| **Table A. Constituents of the total fluid input from 24 hours prior to randomisation until end of day 3. (n=654)** | | |
| --- | --- | --- |
|  | Fluid volumes, ml  Median (IQR) [mean] |  |
| **24 hours prior to randomisation** |  |  |
| Crystalloids | 2000 (1000-3700) [2640] |  |
| Synthetic colloids | 0 (0-500) [312] |  |
| Albumin | 0 (0-0) [52] |  |
| Blood products (RBC, FFP, platelets) | 0 (0-0) [285] |  |
| Other fluids (incl. fluids with medication and nutrition) | 0 (0-868) [551] |  |
| **Day 1** |  |  |
| Trial fluid + open-label trial fluid | 1700 (1000-2850) [2051] |  |
| Crystalloids | 200 (0-1000) [768] |  |
| Synthetic colloids | 0 (0-0) [22] |  |
| Albumin | 0 (0-0) [11] |  |
| Blood products (RBC, FFP, platelets) | 0 (0-0) [207] |  |
| Nutrition | 300 (0-900) [508] |  |
| Fluids with medication | 700 (321-1147) [806] |  |
| Other fluids (incl. water and glucose solutions) | 0 (0-180) [213] |  |
| **Day 2** |  |  |
| Trial fluid + open-label trial fluid | 1500 (500-2500) [1662] |  |
| Crystalloids | 0 (0-500) [455] |  |
| Synthetic colloids | 0 (0-0) [9] |  |
| Albumin | 0 (0-0) [14] |  |
| Blood products (RBC, FFP, platelets) | 0 (0-245) [258] |  |
| Nutrition | 1193 (716-1600) [1168] |  |
| Fluids with medication | 1167 (742-1688) [1256] |  |
| Other fluids (incl. water and glucose solutions) | 0 (0-510) [386] |  |
| **Day 3** |  |  |
| Trial fluid + open-label trial fluid | 500 (0-1100) [773] |  |
| Crystalloids | 0 (0-290) [333] |  |
| Synthetic colloids | 0 (0-0) [5] |  |
| Albumin | 0 (0-0) [12] |  |
| Blood products (RBC, FFP, platelets) | 0 (0-0) [169] |  |
| Nutrition | 1356 (718-1767) [1268] |  |
| Fluids with medication | 967 (515-1585) [1099] |  |
| Other fluids (incl. water and glucose solutions) | 0 (0-440) [319] |  |
| Abbreviations: RBC, red blood cells; FFP, fresh frozen plasma | | |

Unadjusted analyses

| **Table B.** Univariate analyses of associations between patient baseline characteristics and fluid resuscitation volumes given from day 0 to day 3. (n=654) | | |
| --- | --- | --- |
|  | Resuscitation fluid (95% CI)  / ml | P-value  (univariate) |
| Age | 0.2 (-23 – 23) | 0.99 |
| SAPS II | 14 (-5 – 33) | 0.15 |
| Weight | 16 (-1 – 32) | 0.071 |
| Highest lactate | 235 (105 – 364) | 0.0004 |
| Surgery vs. No surgery | 1175 (538 – 1812) | 0.0003 |
| HES vs. Ringer’s | -311 (-926 – 304) | 0.32 |
| SOFA subscores |  |  |
| - Cardiovascular subscore | 408 (207 – 609) | <0.0001 |
| - Renal subscore | 414 (183 – 646) | 0.0005 |
| - Coagulation subscore | 46 (-223 – 314) | 0.74 |
| - Liver subscore | 103 (-298 – 504) | 0.62 |
| - Respiratory subscore | -370 (-683 – -57) | 0.021 |
| Danish hospital | -681 (-1668 – 307) | 0.18 |
| University hospital | -391 (-1006 – 224) | 0.21 |
| Individual trial sites^1^ | NA | <0.0001 |
| ^1^ Only trial sites with at least 25 randomised patients included in the analysis (n=542). Individual SOFA subscores range from 0-4 with 4 being the most severe score. CNS component of the SOFA score was not reported in the 6S trial and not included in the analysis. Abbreviations: HES, hydroxyethyl starch. ICU, intensive care unit. SOFA, Sequential Organ Failure Assessment. | | |

| Complete case analyses  **Table C.** Complete case analyses. Multivariate analyses of associations between hospital characteristics and fluid resuscitation volumes given from day 0 to day 3 adjusted for patient baseline characteristics. (n=479) | | | |
| --- | --- | --- | --- |
|  | Resuscitation fluid (95% CI)  / ml | P-value | |
| **Model A:** |  |  | |
| Danish hospital | -563 (-1840 – 714) | 0.39 | |
| University hospital | -1003 (-1815 – -192) | 0.02 | |
| **Model B:** |  |  | |
| Individual trial sites^1^ | NA | <0.0001 | |
| ^1^Only trial sites with at least 25 patients randomised included (n=397 in 12 trial sites).  Abbreviations: CI, confidence interval. NA, not applicable.  Multivariate generalised linear model A build:  Resuscitation fluid ~ Danish hospital (yes/no) + University hospital (yes/no) + patient baseline characteristics  Multiple generalised linear model B build:  Resuscitation fluid ~ Trial site (as a factor) + patient baseline characteristics | | | |
| **Table D.** Complete case analysis. Multivariate analysis of associations between patient baseline characteristics and fluid resuscitation volumes given from day 0 to day 3. (n=479) | | |  |
|  | Resuscitation fluid (95% CI)  / ml | P-value^1^ |  |
| Age | -11 (-40 – 18) | 0.45 |  |
| SAPS II | 18 (-10 – 46) | 0.21 |  |
| Weight | 20 (1 – 38) | 0.042 |  |
| Highest lactate | 187 (27 – 346) | 0.02 |  |
| Surgery vs. No surgery | 1372 (597 – 2147) | 0.0005 |  |
| HES vs. Ringer’s | -151 (-844 – 541) | 0.67 |  |
| SOFA subscores |  |  |  |
| - Cardiovascular subscore | 392 (134 – 651) | 0.003 |  |
| - Renal subscore | 286 (-5 – 577) | 0.054 |  |
| - Coagulation subscore | 73 (-246 – 392) | 0.65 |  |
| - Liver subscore | 70 (-412 – 552) | 0.78 |  |
| - Respiratory subscore | -618 (-1005 – -230) | 0.002 |  |
| Individual SOFA subscores range from 0-4 with 4 being the most severe score. CNS component of the SOFA score was not reported in the 6S trial and is not included in the analysis.  Abbreviations: CI, confidence interval. HES, hydroxyethyl starch. ICU, intensive care unit. SOFA, Sequential Organ Failure Assessment.  Multivariate generalised linear model build:  Resuscitation fluid ~ SAPS II + age + weight + highest lactate + surgery performed prior to randomisation (yes/no) + allocation (HES/Ringer’s acetate) + cardiovascular SOFA subscore + renal SOFA subscore+ liver SOFA subscore + SOFA subscore + coagulation + respiratory SOFA subscore + trial site (as a factor, with hospital with less than 25 patients grouped) | | |  |

Fluids given after randomisation only

| **Table E.** Multivariate analyses of associations between hospital characteristics and fluid resuscitation volumes given from day 1 to day 3 adjusted for patient baseline characteristics. (n=654) | | |
| --- | --- | --- |
|  | Resuscitation fluids (95% CI)  / ml | P-value |
| **Model A:** |  |  |
| Danish hospital | 566 (-444 – 1577) | 0.27 |
| University hospital | -264 (-758 – 230) | 0.29 |
| **Model B:** |  |  |
| Individual trial sites^1^ | NA | 0.001 |
| ^1^Only trial sites with at least 25 patients randomised included (n=542 in 12 trial sites).  Abbreviations: CI, confidence interval. NA, not applicable.  Multivariate generalised linear model A build:  Resuscitation fluid ~ Danish hospital (yes/no) + University hospital (yes/no) + patient baseline characteristics  Multiple generalised linear model B build:  Resuscitation fluid ~ Trial site (as a factor) + patient baseline characteristics | | |

| **Table F.** Multivariate linear regression analysis of associations between patient baseline characteristics and fluid resuscitation volumes given from day 1 to day 3 adjusted for patient baseline characteristics. (n=654) | | |
| --- | --- | --- |
|  | Resuscitation fluids (95% CI)  / ml | P-value |
| Age | 1 (-20 – 22) | 0.92 |
| SAPS II | 12 (-6 – 31) | 0.19 |
| Weight | 7 (-5 – 20) | 0.24 |
| Highest lactate | 173 (53 – 294) | 0.005 |
| Surgery vs. No surgery | 450 (-73 – 973) | 0.09 |
| HES vs. Ringer’s | -421 (-866 – 24) | 0.06 |
| SOFA subscores |  |  |
| - Cardiovascular subscore | 18 (-130 – 167) | 0.81 |
| - Renal subscore | 212 (9 – 415) | 0.041 |
| - Coagulation subscore | -155 (-358 – 47) | 0.13 |
| - Liver subscore | 12 (-318 – 340) | 0.95 |
| - Respiratory subscore | -219 (-483 – 46) | 0.10 |
| Individual SOFA subscores range from 0-4 with 4 being the most severe score. CNS component of the SOFA score was not reported in the 6S trial and is not included in the analysis.  Abbreviations: CI, confidence interval. HES, hydroxyethyl starch. ICU, intensive care unit. SOFA, Sequential Organ Failure Assessment.  Multivariate generalised linear model build:  Resuscitation fluid ~ SAPS II + age + weight + highest lactate + surgery performed prior to randomisation (yes/no) + allocation (HES/Ringer’s acetate) + cardiovascular SOFA subscore + renal SOFA subscore+ liver SOFA subscore + SOFA subscore + coagulation + respiratory SOFA subscore + trial site (as a factor, with hospital with less than 25 patients grouped) | | |

Discharged and deceased patients included

| **Table G.** Multivariate analyses of associations between hospital characteristics and fluid resuscitation volumes given from day 0 to day 3 adjusted for patient baseline characteristics. Patients who had died or had been discharge within the first 3 days after randomisation included in the analysis. (n=794) | | | |
| --- | --- | --- | --- |
|  | Resuscitation fluids (95% CI)  / ml | P-value | |
| **Model A:** |  |  | |
| Danish hospital | -542 (-1573 – 488) | 0.30 | |
| University hospital | -552 (-1166 – 63) | 0.08 | |
| **Model B:** |  |  | |
| Individual trial sites^1^ | NA | <0.0001 | |
| ^1^Only trial sites with at least 25 patients randomised included (n=662 in 12 trial sites).  Abbreviations: CI, confidence interval. NA, not applicable.  Multivariate generalised linear model A build:  Resuscitation fluid ~ Danish hospital (yes/no) + University hospital (yes/no) + patient baseline characteristics  Multiple generalised linear model B build:  Resuscitation fluid ~ Trial site (as a factor) + patient baseline characteristics | | | |
| **Table H.** Multivariate linear regression analysis of associations between patient baseline characteristics and fluid resuscitation volumes given from day 0 to day 3 adjusted for patient baseline characteristics. Patients who had died or had been discharge within the first 3 days after randomisation included in the analysis. (n=794) | | |  |
|  | Resuscitation fluids (95% CI)  / ml | P-value^1^ |  |
| Age | -5 (-29 – 19) | 0.66 |  |
| SAPS II | 7 (-17 – 31) | 0.54 |  |
| Weight | 13 (-0.5 – 27) | 0.058 |  |
| Highest lactate | 75 (-21 – 171) | 0.13 |  |
| Surgery vs. No surgery | 1209 (619 – 1798) | <0.0001 |  |
| HES vs. Ringer’s | -250 (-769 – 269) | 0.35 |  |
| SOFA subscores |  |  |  |
| - Cardiovascular subscore | 383 (204 – 563) | <0.0001 |  |
| - Renal subscore | 261 (28 – 495) | 0.03 |  |
| - Coagulation subscore | 65 (-204 – 334) | 0.63 |  |
| - Liver subscore | -88 (-452 – 276) | 0.64 |  |
| - Respiratory subscore | -327 (-623 – -30) | 0.03 |  |
| Individual SOFA subscores range from 0-4 with 4 being the most severe score. CNS component of the SOFA score was not reported in the 6S trial and is not included in the analysis.  Abbreviations: CI, confidence interval. HES, hydroxyethyl starch. ICU, intensive care unit. SOFA, Sequential Organ Failure Assessment.  Multivariate generalised linear model build:  Resuscitation fluid ~ SAPS II + age + weight + highest lactate + surgery performed prior to randomisation (yes/no) + allocation (HES/Ringer’s acetate) + cardiovascular SOFA subscore + renal SOFA subscore+ liver SOFA subscore + SOFA subscore + coagulation + respiratory SOFA subscore + trial site (as a factor, with hospital with less than 25 patients grouped) | | |  |

Performing analyses using mixed model with trial site as a random effect

| **Table I.** Mixed model analysis of associations between individual trial sites and fluid resuscitation volumes given from day 0 to day 3 adjusted for patient baseline characteristics. (n=542) | | | | |
| --- | --- | --- | --- | --- |
|  | Resuscitation fluids (95% CI)  / ml | P-value | | |
| Individual trial sites^1^ | NA | 0.03 | | |
| ^1^Only trial sites with at least 25 patients randomised included (n=542 in 12 trial sites).  Abbreviations: CI, confidence interval. NA, not applicable.  Mixed model:  Resuscitation fluid ~ Trial site (as a random-effects factor) + patient baseline characteristics | | | | |
| **Table J.** Mixed model analysis of associations between patient baseline characteristics and fluid resuscitation volumes given from day 0 to day 3 adjusted for patient baseline characteristics. (n=654) | | | |  |
|  | Resuscitation fluids (95% CI)  / ml | | P-value^1^ |  |
| Age | -7 (-31 – 17) | | 0.58 |  |
| SAPS II | 11 (-14 – 35) | | 0.40 |  |
| Weight | 14 (-2 – 31) | | 0.08 |  |
| Highest lactate | 191 (60 – 322) | | 0.004 |  |
| Surgery vs. No surgery | 1389 (742 – 2036) | | <0.0001 |  |
| HES vs. Ringer’s | -275 (-851 – 302) | | 0.35 |  |
| SOFA subscores |  | |  |  |
| - Cardiovascular subscore | 379 (166 – 593) | | 0.0005 |  |
| - Renal subscore | 269 (25 – 513) | | 0.03 |  |
| - Coagulation subscore | 73 (-200 – 347) | | 0.60 |  |
| - Liver subscore | -49 (-440 – 342) | | 0.81 |  |
| - Respiratory subscore | -395 (-714 – -76) | | 0.02 |  |
| Individual SOFA subscores range from 0-4 with 4 being the most severe score. CNS component of the SOFA score was not reported in the 6S trial and is not included in the analysis.  Abbreviations: CI, confidence interval. HES, hydroxyethyl starch. ICU, intensive care unit. SOFA, Sequential Organ Failure Assessment.  Multivariate generalised linear model build:  Resuscitation fluid ~ SAPS II + age + weight + highest lactate + surgery performed prior to randomisation (yes/no) + allocation (HES/Ringer’s acetate) + cardiovascular SOFA subscore + renal SOFA subscore+ liver SOFA subscore + SOFA subscore + coagulation + respiratory SOFA subscore + trial site (as a random-effects factor, with hospital with less than 25 patients grouped) | | | |  |

Excluding patients who were withdrawn from the intervention during the first 3 days after randomisation

| **Table K.** Multivariate analyses of associations between hospital characteristics and fluid resuscitation volumes given from day 0 to day 3 adjusted for patient baseline characteristics. Patients who were withdrawn from the intervention during the first 3 days were excluded (n=561). | | | | |
| --- | --- | --- | --- | --- |
|  | Resuscitation fluids (95% CI)  / ml | P-value | | |
| **Model A:** |  |  | | |
| Danish hospital | -427 (-1612 – 758) | 0.48 | | |
| University hospital | -807 (-1488 – -126) | 0.02 | | |
| **Model B:** |  |  | | |
| Individual trial sites^1^ | NA | <0.0001 | | |
| ^1^Only trial sites with at least 25 patients randomised included (n=465 in 12 trial sites).  Abbreviations: CI, confidence interval. NA, not applicable.  Multivariate generalised linear model A build:  Resuscitation fluid ~ Danish hospital (yes/no) + University hospital (yes/no) + patient baseline characteristics  Multiple generalised linear model B build:  Resuscitation fluid ~ Trial site (as a factor) + patient baseline characteristics | | | | |
| **Table L.** Multivariate analysis of associations between patient baseline characteristics and fluid resuscitation volumes given from day 0 to day 3 adjusted for patient baseline characteristics. Patients who were withdrawn from the intervention during the first 3 days were excluded (n=561). | | | |  |
|  | Resuscitation fluids (95% CI)  / ml | | P-value^1^ |  |
| Age | 3 (-23 – 29) | | 0.84 |  |
| SAPS II | 14 (-12 – 41) | | 0.28 |  |
| Weight | 15 (-2 – 31) | | 0.08 |  |
| Highest lactate | 212 (60 – 364) | | 0.006 |  |
| Surgery vs. No surgery | 1300 (624 – 1976) | | 0.0002 |  |
| HES vs. Ringer’s | -181 (-769 – 406) | | 0.55 |  |
| SOFA subscores |  | |  |  |
| - Cardiovascular subscore | 420 (225 – 615) | | <0.0001 |  |
| - Renal subscore | 265 (-10 – 541) | | 0.06 |  |
| - Coagulation subscore | -107 (-417 – 202) | | 0.50 |  |
| - Liver subscore | -95 (-536 – 345) | | 0.67 |  |
| - Respiratory subscore | -491 (-847 – -135) | | 0.007 |  |
| Individual SOFA subscores range from 0-4 with 4 being the most severe score. CNS component of the SOFA score was not reported in the 6S trial and is not included in the analysis.  Abbreviations: CI, confidence interval. HES, hydroxyethyl starch. ICU, intensive care unit. SOFA, Sequential Organ Failure Assessment.  Multivariate generalised linear model build:  Resuscitation fluid ~ SAPS II + age + weight + highest lactate + surgery performed prior to randomisation (yes/no) + allocation (HES/Ringer’s acetate) + cardiovascular SOFA subscore + renal SOFA subscore+ liver SOFA subscore + SOFA subscore + coagulation + respiratory SOFA subscore + trial site (as a factor, with hospital with less than 25 patients grouped) | | | |  |
